# Clear-Cell Mesothelioma of Uterine Corpus: Diagnostic Challenges in Intraoperative Frozen Sections

**DOI:** 10.3390/diagnostics13061119

**Published:** 2023-03-15

**Authors:** Tip Pongsuvareeyakul, Kanokkan Saipattranusorn, Kornkanok Sukpan, Prapaporn Suprasert, Surapan Khunamornpong

**Affiliations:** 1 Department of Pathology, Faculty of Medicine, Chiang Mai University, Chiang Mai 50200, Thailand; 2 Department of Pathology, Chonburi Hospital, Chonburi 20000, Thailand; 3 Department of Obstetrics and Gynecology, Faculty of Medicine, Chiang Mai University, Chiang Mai 50200, Thailand

**Keywords:** clear-cell mesothelioma, peritoneum, clear-cell carcinoma, frozen section, uterus

## Abstract

The clear-cell variant of epithelioid mesothelioma is an extremely rare neoplasm of the peritoneum. It shares histomorphologic features overlapping with a wide variety of tumors including carcinomas and other non-epithelial neoplasms. The diagnosis of peritoneal clear-cell mesothelioma is not always straightforward, despite known immunohistochemistry (IHC) markers. Due to its rarity, this entity may be diagnostically confused with other clear-cell neoplasms, particularly in intraoperative frozen sections. Here, we present a case of clear-cell mesothelioma originating in the uterine serosa that was initially misdiagnosed as clear-cell adenocarcinoma in the intraoperative frozen section. Microscopically, the tumor showed diffuse tubulocystic spaces of variable size lined by clear cells with moderate nuclear atypia. Immunohistochemical staining confirmed the diagnosis of clear-cell mesothelioma. Recognition of this entity, albeit rare, is important as the diagnosis may significantly affect the management considerations. The judicious use of an IHC panel helps to distinguish this tumor from other mimickers.

**Table 1:** Malignant mesothelioma of the peritoneum in women is 3.5 times less common than the pleural counterpart [1]. The incidence rate of peritoneal mesothelioma is only 0.1 per 100,000 women [2]. The differential diagnosis of peritoneal mesothelioma includes a wide variety of malignant tumors in contrast to pleural mesothelioma, which should be distinguished from pulmonary adenocarcinoma in the majority of cases [3]. Peritoneal mesothelioma has clinical, morphological, and molecular features that are distinctive from the pleural counterpart [4] (this table). It occurs more commonly in young women, and the association with asbestos exposure is much less strong than that of pleural mesothelioma [4]. In a large study of 164 cases of peritoneal mesothelioma in women [2], 40 cases (24.4%) had an initial referral diagnosis of Mullerian-type carcinomas or non-gynecological carcinomas. The large majority of tumors had epithelioid morphology (80.5%), whereas the remainder (19.5%) had biphasic morphology (epithelioid and sarcomatoid). Mixed architectural patterns were observed in 92% of cases, including papillary, solid, tubular or glandular, single-cell, and cystic patterns. The presence of clear cells or hobnail cells was found in a minority of cases (number not specified) [2]. Clear-cell mesothelioma represents an extremely rare subtype of peritoneal mesothelioma [5]. As the tumor shows clear cytoplasmic features, the histomorphology is overlapping with Mullerian clear-cell adenocarcinoma of the female genital organs. In this report, we describe a case of clear-cell mesothelioma originating in the uterine serosa. The morphological features of this tumor closely mimic clear-cell adenocarcinoma, resulting in an incorrect intraoperative frozen section diagnosis, which affected the surgical management decision. Comparison of clinico-pathological features of mesothelioma [1,2,4,6,7,8,9,10,11,12,13,14,15].

Clinico-Pathological Features	Pleural Mesothelioma	Peritoneal Mesothelioma	Peritoneal Clear-Cell Mesothelioma
Estimated incidence (per 100,000 population)	0.6–0.9	0.1	Extremely rare
Male	1.1–1.9	0.1	
Female	0.2–0.3	0.1	
Male: Female ratio	3.9:1	1.3:1	Female
Median age (years)	65–70	49–69 (49 in female) a	Limited data
Association with asbestos exposure	70%	33% (5–23% in female) a	Limited data
Association with germline cancer susceptibility mutations	7%	25%	Limited data
Histologic patterns	Epithelioid (55%) Biphasic (20%) Sarcomatoid (10%)	Epithelioid (82%) Biphasic (13%) Sarcomatoid (5%)	Epithelioid (limited data)
Loss of BAP1 immunoexpression b	80%a (especially epithelioid type)	57% a	Limited data
Median overall survival time (months)	17–20	53	Limited data

^a^ Data in female patients. ^b^ Surrogate marker for BAP1 mutation.

**Figure 1:**
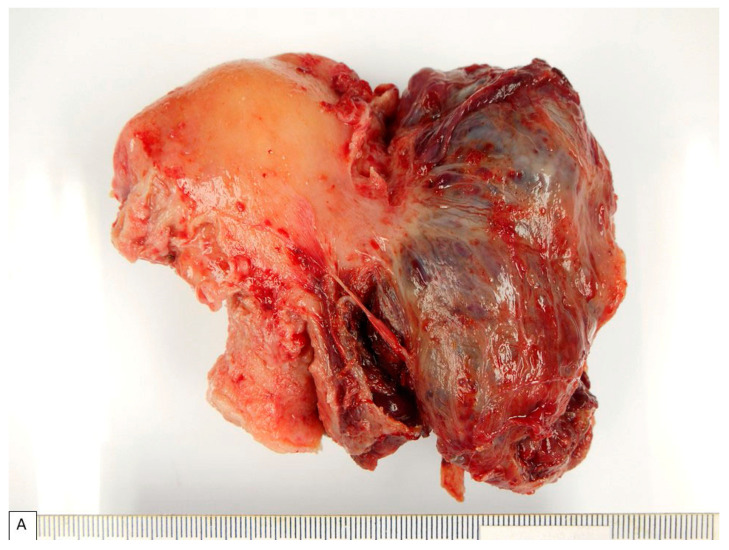

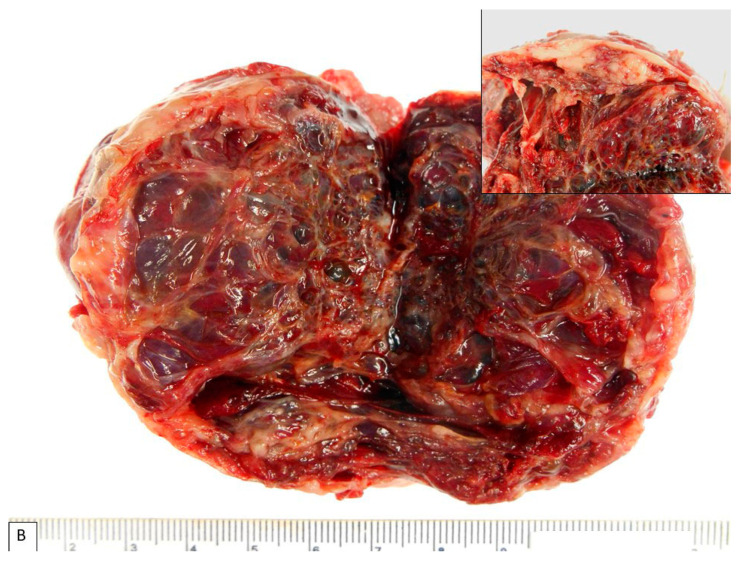
A 28-year-old Thai pregnant woman underwent an uneventful cesarean section at a local hospital. A 6 cm mass, right lateral to the uterus, was incidentally found. Follow-up computed tomography of the whole abdomen showed a 9.1 cm cystic mass with irregularly thick septation in the right adnexal region, consistent with an ovarian tumor. The patient was referred to Maharaj Nakorn Chiang Mai Hospital for further management two months after delivery. The patient had no clinical history of asbestos exposure or underlying diseases. Her family history was unremarkable. Preoperative laboratory investigations revealed a slight elevation of the CA125 tumor marker (70.6 U/mL; normal 0–35 U/mL). Intraoperatively, a large tumor protruding from uterine serosa was seen. Total abdominal hysterectomy was performed, and the uterus was sent for an intraoperative frozen section. Macroscopically, a 9 × 7 × 5.5 cm serosal tumor was found at the right posterolateral wall, as presented in (**A**). Cut surface of the tumor was dark red and revealed predominantly thin-walled multicystic to spongy tissue containing blood, as presented in (**B**), with focal tan solid area at the periphery ((**B**), **inset**). Frozen section diagnosis was clear-cell adenocarcinoma involving uterine serosa. Then, the patient underwent a complete surgical staging procedure, including bilateral salpingo-oophorectomy (BSO), bilateral pelvic node biopsy, omental biopsy, and peritoneal washing. Such intraoperative diagnosis was the main reason for complete surgical staging including immediate BSO and lymphadenectomy, which may not be the necessary surgical procedure for mesothelioma in young women in whom preservation of ovarian function is of concern. This figure and Figure 2 demonstrate the macroscopic and microscopic appearance of clear-cell mesothelioma that has led to the intraoperative diagnosis of clear-cell adenocarcinoma. The occurrence of extragenital clear-cell adenocarcinoma, although uncommon, could be expected, as clear-cell adenocarcinoma is common in the Eastern world, accounting for up to 27% of ovarian epithelial cancers in Japan [6], and this tumor can arise from non-ovarian endometriosis. While the diagnosis of clear-cell adenocarcinoma in the endometrium or the ovary may not require or depend on immunohistochemistry, the diagnosis of clear-cell adenocarcinoma of peritoneal origin needs to be supported by an appropriate and extensive immunohistochemical panel. The differential diagnoses of clear-cell mesothelioma include Mullerian-type adenocarcinomas with clear-cell features, non-Mullerian clear-cell carcinomas with relatively occult origin (e.g., clear-cell renal cell carcinoma), and other non-epithelial clear-cell neoplasms.

**Figure 2:**
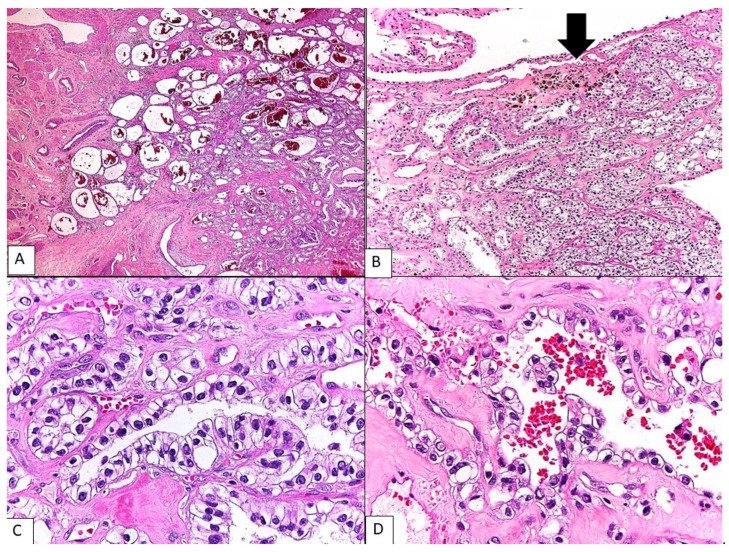
Histologic findings of clear-cell mesothelioma. The tumor showed an infiltrative border involving the outer myometrial wall (**A**) (**H&E**, **40×**). The tumor was composed of diffuse tubulocystic spaces of varying size lined by monotonously uniform clear cells (**B**) (**H&E**, **100×**). These cells exhibited abundant clear cytoplasm and hyperchromatic nuclei with moderate atypia and occasional distinct nucleoli (**C**) (**H&E**, **400×**) or had a hobnail appearance (**D**) (**H&E**, **400×**). Mitoses were rare (<1 in 10 high power fields). Scattered hemorrhage and hemosiderin deposits were present in fibrous septa ((**B**), **arrow**), but endometriosis was not identified. The tubulocystic pattern of this tumor is indistinguishable from that of clear-cell adenocarcinoma. However, the tumor lacks other common features of clear-cell adenocarcinoma including papillary architecture, hyalinized stromal material, adenofibromatous component, and the association with endometriotic focus. The absence of these features and the extraovarian location led to serious consideration for other tumors with clear-cell morphology. In the absence of a clinically identifiable primary site in any visceral organs, the exclusion of mesothelioma is necessary.

**Figure 3:**
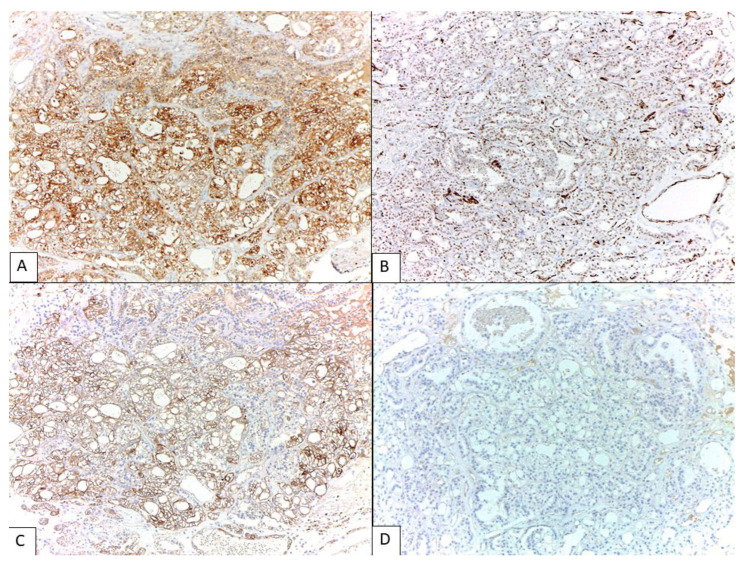
Immunohistochemical profile of clear-cell mesothelioma. Diffuse immunopositivity was observed for calretinin (**A**), (100×), Wilms’ tumor-1 (WT-1) (**B**), (100×), cytokeratin (CK) 5/6 (**C**), (100×), and vimentin. There was focal staining of epithelial membrane antigen (EMA) and CD10 (non-luminal pattern), whereas the staining for epithelial-cell adhesion molecule (Ep-CAM, BerEP4) (**D**)), (100×), PAX8, Napsin-A, estrogen receptor (ER), renal cell carcinoma antibody (RCC), inhibin, GATA3, and thyroid transcription factor-1 (TTF-1) was negative. The tumor exhibited wild-type expression of p53. Ki-67 proliferative index was 5–10% of cells. The panel of immunohistochemical stains, including the markers supporting mesothelial origin (e.g., calretinin, CK5/6, podoplanin (D2-40), WT1, mesothelin, Hector Battifora mesothelial-1 (HBME-1), or thrombomodulin) and the markers for non-mesothelial neoplasms, is usually helpful in distinguishing mesothelioma from other tumors with similar morphology. It is important to note that if mesothelioma is not considered in the list of differential diagnosis and mesothelial markers are not included, the immunoprofile of mesothelioma can partially overlap with clear-cell adenocarcinoma or other Mullerian-type adenocarcinomas with clear-cell-like features. Mullerian-type adenocarcinomas and mesothelioma are positive for CK7 and EMA. Although negativity for PAX8 and Napsin A is unusual for clear-cell adenocarcinoma, it should be noted that some peritoneal mesotheliomas are positive for PAX8 [8] and Napsin A may be negative in almost 30% of clear-cell adenocarcinomas [16]. WT-1 positivity, as seen in mesothelioma, is characteristic of Mullerian-type serous adenocarcinoma but is unusual for clear-cell adenocarcinoma. Negativity for ER and wild-type p53 pattern is similarly observed in most clear-cell adenocarcinoma and mesothelioma, whereas ER expression is common in serous and endometrioid adenocarcinomas, and abnormal p53 pattern is characteristic of high-grade serous adenocarcinoma [10]. Current recommendations to distinguish mesothelioma from carcinoma is the immunoreactivity for two mesothelial markers and shows negativity for two epithelial carcinoma markers [3]. In this patient, the immunoprofile of calretinin, CK5/6, BerEp4, and PAX8 help exclude clear-cell adenocarcinoma. The location of the tumor in the posterolateral aspect of uterus should also raise the differential diagnosis of rare tumors with mesonephric-related origin, mesonephric carcinoma and Wolffian tumor of uterine ligament. The tumors in this group share similar calretinin positivity. Mesonephric carcinoma is immunopositive for PAX8, GATA3, TTF1 and CD10 (luminal pattern), whereas Wolffian tumor is mostly negative with PAX8 and GATA3. Wolffian tumor is also positive for CK7 and sex cord-stromal markers such as inhibin and FOXL2 [17]. This figure shows the immunohistochemical stains that confirm the diagnosis of clear-cell mesothelioma.

**Figure 4:**
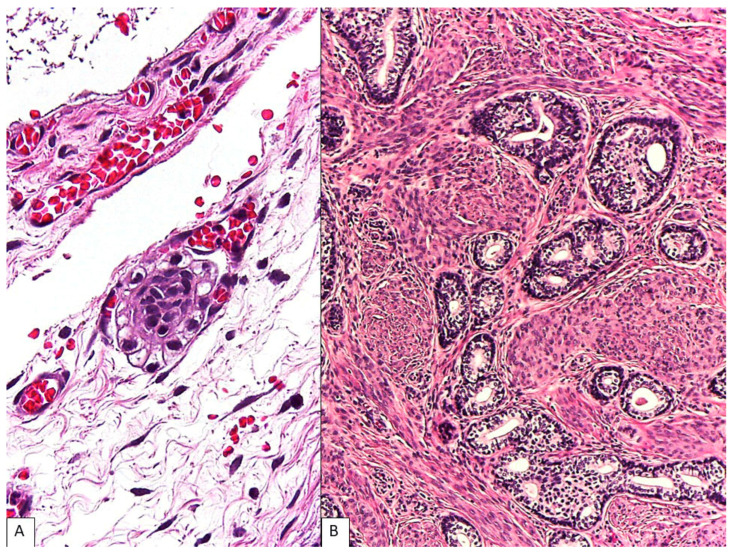
The additional findings in uterus and both adnexa. A cluster of atypical clear cells (less than 0.2 mm) was identified on the surface of left ovary, consistent with metastatic clear-cell mesothelioma (**A**) (**H&E**, **400×**). The cervix showed an incidental adenoid basal carcinoma in the right lateral wall (**B**) (**H&E**, **100×**), measuring 4.5 mm in depth and without lymphovascular involvement (FIGO stage IA2). No neoplastic lesion was seen in the corpus, right ovary, bilateral fallopian tubes, pelvic lymph nodes, omentum, and peritoneal washing cytology. The patient received six cycles of adjuvant chemotherapy and hormonal replacement therapy. She remained well without evidence of disease 12 months after surgery. This figure represents the additional findings after extensive histologic examination of the uterus and both adnexa. A single metastatic focus of the tumor was identified in the contralateral ovarian surface (**A**). In general, localized mesothelioma appears to have a more favorable clinical course than diffuse mesothelioma [6,18]. The prognosis of peritoneal mesothelioma could not be predicted by histomorphology alone, although deciduoid morphology or nuclear grade 3 has been reported to be an independent unfavorable prognostic predictor [2]. Based on NCCN guidelines 2022, epithelioid subtype, Ki-67 index < 9%, peritoneal cancer index of 17 or less, absence of lymph node involvement, and complete cytoreduction are favorable prognostic features [7]. More recently, a novel VHL gene mutation has been reported in peritoneal clear-cell mesothelioma with an indolent clinical behavior [5]. Given the rarity of this entity, the clinical course, prognostic predictors, pathogenesis, and molecular profiles remain to be clarified. This patient had co-existing adenoid basal carcinoma of the uterine cervix (**B**). In a recent series of 18 women with localized peritoneal mesothelioma, a history of other cancers (breast, endometrial, and ovarian) was found in 4 of 13 cases (31%) with available data [6]. Family history available in 11 patients also noted the presence of cancer in their family members in all cases, 82% of which were first-degree relatives. In women with peritoneal mesothelioma, the presence of another tumor should also be monitored [2,6]. In conclusion, clear-cell mesothelioma represents a diagnostic challenge for pathologists. Recognition of this entity, albeit rare, is important as the diagnosis may significantly affect the management considerations.

## Data Availability

The data of this report are available from the corresponding authors upon request.

## References

[1] Pavlisko E.N., Liu B., Green C., Sporn T.A., Roggli V.L. (2020). Malignant Diffuse Mesothelioma in Women: A Study of 354 Cases. Am. J. Surg. Pathol..

[2] Malpica A., Euscher E.D., Marques-Piubelli M.L., Ferrufino-Schmidt M.C., Miranda R.N., Sams R., Royal R.E., Raghav K.P.S., Fournier K.F., Ramalingam P. (2021). Malignant Mesothelioma of the Peritoneum in Women: A Clinicopathologic Study of 164 Cases. Am. J. Surg. Pathol..

[3] Husain A.N., Colby T.V., Ordonez N.G., Allen T.C., Attanoos R.L., Beasley M.B., Butnor K.J., Chirieac L.R., Churg A.M., Dacic S. (2018). Guidelines for Pathologic Diagnosis of Malignant Mesothelioma 2017 Update of the Consensus Statement from the International Mesothelioma Interest Group. Arch. Pathol. Lab. Med..

[4] Chapel D.B., Schulte J.J., Absenger G., Attanoos R., Brcic L., Butnor K.J., Chirieac L., Churg A., Galateau-Salle F., Hiroshima K. (2021). Malignant peritoneal mesothelioma: Prognostic significance of clinical and pathologic parameters and validation of a nuclear-grading system in a multi-institutional series of 225 cases. Mod. Pathol..

[5] Smith-Hannah A., Naous R. (2019). Primary peritoneal epithelioid mesothelioma of clear cell type with a novel VHL gene mutation: A case report. Hum. Pathol..

[6] Malpica A., Euscher E.D., Marques-Piubelli M.L., Miranda R.N., Raghav K.P., Fournier K.F., Ramalingam P. (2022). Localized Malignant Peritoneal Mesothelioma (LMPeM) in Women: A Clinicopathologic Study of 18 Cases. Am. J. Surg. Pathol..

[7] Singhi A.D., Krasinskas A.M., Choudry H.A., Bartlett D.L., Pingpank J.F., Zeh H.J., Luvison A., Fuhrer K., Bahary N., Seethala R.R. (2016). The prognostic significance of BAP1, NF2, and CDKN2A in malignant peritoneal mesothelioma. Mod. Pathol..

[8] National Comprehensive Cancer Network Mesothelioma: Peritoneal (Version:1.2023). https://www.nccn.org/professionals/physician_gls/pdf/meso_peritoneal.pdf.

[9] National Comprehensive Cancer Network Mesothelioma: Pleural (Version: 1.2023). https://www.nccn.org/professionals/physician_gls/pdf/meso_pleural.pdf.

[10] Centers for Disease Control and Prevention Incidence of Malignant Mesothelioma, 1999–2018. https://www.cdc.gov/cancer/uscs/about/data-briefs/no27-incidence-malignant-mesothelioma-1999-2018.htm.

[11] Mao W., Zhang X., Guo Z., Gao Z., Pass H.I., Yang H., Carbone M. (2017). Association of Asbestos Exposure with Malignant Mesothelioma Incidence in Eastern China. JAMA Oncol..

[12] Sugarbaker P.H., Welch L.S., Mohamed F., Glehen O. (2003). A review of peritoneal mesothelioma at the Washington Cancer Institute. Surg. Oncol. Clin. N. Am..

[13] Zhuo M., Zheng Q., Chi Y., Jia B., Zhao J., Wu M., An T., Wang Y., Li J., Zhao X. (2019). Survival analysis via nomogram of surgical patients with malignant pleural mesothelioma in the Surveillance, Epidemiology, and End Results database. Thorac. Cancer.

[14] Chapel D.B., Hornick J.L., Barlow J., Bueno R., Sholl L.M. (2022). Clinical and molecular validation of BAP1, MTAP, P53, and Merlin immunohistochemistry in diagnosis of pleural mesothelioma. Mod. Pathol..

[15] Panou V., Gadiraju M., Wolin A., Weipert C.M., Skarda E., Husain A.N., Patel J.D., Rose B., Zhang S.R., Weatherly M. (2018). Frequency of Germline Mutations in Cancer Susceptibility Genes in Malignant Mesothelioma. J. Clin. Oncol.

[16] Weidemann S., Bohle J.L., Contreras H., Luebke A.M., Kluth M., Buscheck F., Hube-Magg C., Hoflmayer D., Moller K., Fraune C. (2021). Napsin A Expression in Human Tumors and Normal Tissues. Pathol. Oncol. Res..

[17] Gilk B. (2020). WHO Classification of Femal Genital Tumours.

[18] Marchevsky A.M., Khoor A., Walts A.E., Nicholson A.G., Zhang Y.Z., Roggli V., Carney J., Roden A.C., Tazelaar H.D., Larsen B.T. (2020). Localized malignant mesothelioma, an unusual and poorly characterized neoplasm of serosal origin: Best current evidence from the literature and the International Mesothelioma Panel. Mod. Pathol..

